# Genetic Divergence and Chemotype Diversity in the Fusarium Head Blight Pathogen *Fusarium poae*

**DOI:** 10.3390/toxins9090255

**Published:** 2017-08-23

**Authors:** Adriaan Vanheule, Marthe De Boevre, Antonio Moretti, Jonathan Scauflaire, Françoise Munaut, Sarah De Saeger, Boris Bekaert, Geert Haesaert, Cees Waalwijk, Theo van der Lee, Kris Audenaert

**Affiliations:** 1 Department of Applied Biosciences, Faculty of Bioscience Engineering, Ghent University, 9000 Ghent, Belgium; Adriaan.vanheule@ugent.be (A.V.); Boris.bekaert@ugent.be (B.B.); Geert.haesaert@ugent.be (G.H.); 2 Laboratory of Applied Mycology and Phenomics, Department of Applied Biosciences, Faculty of Bioscience Engineering, Ghent University, 9000 Ghent, Belgium; 3 Department of Bioanalysis, Faculty of Pharmaceutical Sciences, Ghent University, 9000 Ghent, Belgium; Marthe.deboevre@ugent.be; 4 Institute of Sciences of Food Production, National Research Council, 70126 Bari, Italy; antonio.moretti@ispa.cnr.it; 5 Applied Microbiology, Earth and Life Institute, Université Catholique de Louvain, 1348 Louvain-la-Neuve, Belgium; Jonathan.scauflaire@uclouvain.be (J.S.); francoise.munaut@outlook.com (F.M.); 6 Wageningen University and Research Centre, 6708PB Wageningen, The Netherlands; cees.waalwijk@wur.nl (C.W.); theo.vanderlee@wur.nl (T.v.d.L.)

**Keywords:** *Fusarium*, AFLP, trichothecenes, transposable element, mating type, meiosis

## Abstract

Fusarium head blight is a disease caused by a complex of *Fusarium* species. *F. poae* is omnipresent throughout Europe in spite of its low virulence. In this study, we assessed a geographically diverse collection of *F. poae* isolates for its genetic diversity using AFLP (Amplified Fragment Length Polymorphism). Furthermore, studying the mating type locus and chromosomal insertions, we identified hallmarks of both sexual recombination and clonal spread of successful genotypes in the population. Despite the large genetic variation found, all *F. poae* isolates possess the nivalenol chemotype based on Tri7 sequence analysis. Nevertheless, *Tri* gene clusters showed two layers of genetic variability. Firstly, the Tri1 locus was highly variable with mostly synonymous mutations and mutations in introns pointing to a strong purifying selection pressure. Secondly, in a subset of isolates, the main trichothecene gene cluster was invaded by a transposable element between *Tri5* and *Tri6*. To investigate the impact of these variations on the phenotypic chemotype, mycotoxin production was assessed on artificial medium. Complex blends of type A and type B trichothecenes were produced but neither genetic variability in the *Tri* genes nor variability in the genome or geography accounted for the divergence in trichothecene production. In view of its complex chemotype, it will be of utmost interest to uncover the role of trichothecenes in virulence, spread and survival of *F. poae*.

## 1. Introduction

The genus *Fusarium* is implicated in devastating plant diseases. Of primary importance is the *sambucinum* species complex, which contains species causing Fusarium Head Blight (FHB) on a number of small-grain cereals such as wheat [1]. The economic impact of this disease is exacerbated by the deposition in the cereal matrix of mycotoxins, which ultimately end up in the food and feed chain with significant health risks for humans and animals [2]. Many FHB-associated species may co-occur on the host and the composition of this population is variable throughout and between growing seasons [3]. *F. graminearum* is the predominant species within the FHB complex [1]. In addition, *F. poae*, has been described as one of the most prominent FHB species in several studies [4,5,6,7]. Its occurrence is often correlated with the incidence of other *Fusarium* species and its preponderance increased with the application of azole fungicides [4,8,9].

The most important class of mycotoxins associated with FHB is the trichothecenes. They are produced by a large number of *Fusarium* species and can be divided mainly into type A (diacetoxyscirpenol (DAS), neosolaniol (NEO), and T-2 toxin, HT-2 toxin) and type B trichothecenes (deoxynivalenol (DON), nivalenol (NIV) and fusarenon-x (FUS-X), differing at the functional group occupying the carbon 8 position [10]. Genes for trichothecene biosynthesis are organized in different loci and clusters. Homologs with different functionality in different *Fusarium* species determine which end products will be produced [11]. The main determinant for type A vs. type B production resides in different alleles of the *Tri1* gene [12]. Surprisingly however, several authors have simultaneously detected both type A and type B trichothecenes from isolates of *F. poae* [13,14,15].

The specific lifestyle of fungal pathogens greatly influences their epidemiology, and their potential for adaptation and evolution. Species that combine sexual and asexual reproduction, and that harbor active transposable elements (TEs) have been theorized to possess the greatest potential for genetic variation and hence pose the highest threat to durable disease management [16]. The occurrence of “cryptic” stages may complicate the identification of the precise lifestyle of fungi. This is the case when a sexual cycle is presumed to occur, but has never been witnessed, such as observed for several *Fusarium* species [17]. Recombination is a clear indication that a sexual cycle is ongoing in fungi [18], and genome-driven discovery of meiosis-specific markers may uncover the footprints of a sexual cycle [19]. The occurrence of repeat-induced point mutation (RIP), a defense mechanism against TEs, is another line of evidence for meiosis, as it only occurs during the pre-meiotic stage [20]. Subsequently, degenerated remnants of TE copies remain in the genome after RIP passage [20]. The activity of RIP has recently been extensively proven for *F. poae* [19]. The mating locus (MAT1) is another important genetic fingerprint for a sexual cycle. This locus can be occupied by two idiomorphs MAT1-1 and MAT1-2 and the equal distribution of both idiomorphs in fungal populations has been considered as an indication of an active sexual cycle. The occurrence of both MAT1-1 and MAT1-2 has been confirmed for *F. poae* isolates [17,21].

One of the benefits of sexual reproduction is meiosis, which introduces diversity via recombination. Many studies have been carried out which investigate the population diversity of *F. poae*. Kerenyi et al. [22] detected a highly complicated composition of vegetative compatibility groups (VCGs) in 50 geographically diverse isolates. Somma et al. [23] found a high level of intra-species variability in a geographically narrow population using amplified fragment length polymorphism (AFLP) [24], which was also found for a worldwide *F. poae* collection [21,25] with AFLP and inter simple sequence repeats (ISSR). Consistently, geographic origin could only partially explain this large diversity [22].

Contrary to the results in genome-wide approaches, multi-locus barcoding consistently leads to the detection of rather low intra-species variability in *F. poae* [26,27]. This discrepancy may be in part explained by the specific genome composition of *F. poae*. Isolates of this species have four core chromosomes (the “core genome”) and a set of supernumerary chromosomes, highly variable in size, composition, and number between individuals [19,28]. It has been shown that their presence leads to significant rearrangements within the core chromosomes [19]. Two major translocations from the supernumerary genome into core chromosome 3 of *F. poae* isolate 2516 were found, each exceeding several 100 kb. Finally, the supernumerary genome harbors numerous active TEs (where they are protected from RIP), which can migrate from the supernumerary to the core genome.

Species that are capable of combining both sexual and asexual reproduction, and species that possess an active arsenal of TEs, are considered to be the biggest challenge for durable disease management [16] and a key trait of a successful pathogens [29]. We set out to investigate both features in this study. We started by assessing the genetic diversity using a genome wide approach in a representative *F. poae* collection from different geographic origins. We searched for hallmarks of sexual and asexual reproduction, and indications for how genomes evolve in a population. In addition, we investigated the genetic variability in the trichothecene gene clusters and tried to link this genetic variability with the highly variable mycotoxin profiles of *F. poae* comprising both type A and type B trichothecenes.

## 2. Results

### 2.1. Genetic Diversity of Fusarium poae

For this study, we used 69 available *F. poae* isolates (Table 1) and AFLP analysis was performed to assess their genetic diversity. A total of 247 markers were scored of which 201 were polymorphic. As a control *F. poae* isolate 9125 was scored independently three times in the analysis. The clustering of these three fingerprints shows the reproducibility of the AFLP method but also illustrates the variability obtained for a perfectly clonal isolate. The genotyping error rate calculated from these three repetitions was 3.3–3.8% similar to levels described in literature (2–5%) [30,31].

**Figure 1:**
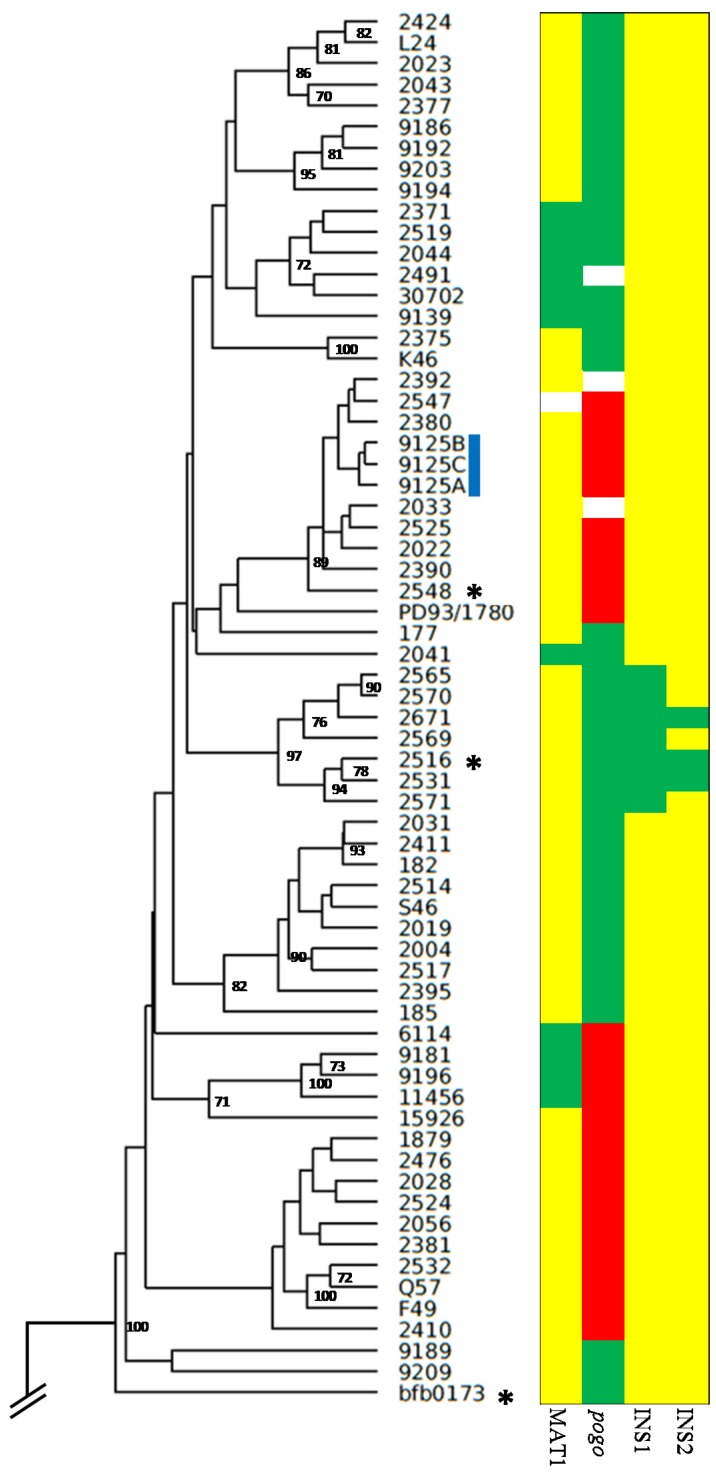
AFLP tree of *F. poae* isolates, and superposition of genetic markers. UPGMA (Unweighted Pair Group Method with Arithmetic Mean) cluster analysis was performed based on the Dice similarity coefficients between the isolates. In total, 247 markers were scored of which 201 were polymorphic. Three technical replicates of isolate 9125 are also included in the graph (blue bar). Clusters of isolates that share more than 90% genetic similarity according to the Dice similarity coefficient are grouped with the same colors. Bootstrap values exceeding 70% are given at the nodes, based on 1000 replications. Four genetic markers that were determined throughout this study are listed (*MAT1*, *pogo*, INS1, and INS2). White entries are markers for which no data could be collected. *MAT1*: yellow is MAT1-1, green is MAT1-2. *Pogo*: green indicates presence of the *pogo* element between *Tri5* and *Tri6* in the major gene cluster, red indicates absence. INS1 and INS2 are the major insertions of supernumerary sequence into chromosome 3 of a subset of isolates. Yellow indicates the absence of the insertion, green indicates the presence of the insertion. The isolate for which reference genomes is available is shown with asterisks.

Based on the AFLP analysis, the *F. poae* isolates are divided into sub-clusters with no clear correspondence to either host or geographic origin. This is exemplified by isolates from maize (L24, K46, S46, Q57, F49), isolates from barley (6114, 175, 182, 185, bfb0176) and isolates from Italy (designation 9###) which are scattered throughout the tree. However, the only Chinese isolate in the study (bfb0173) is clearly distinct from all other *F. poae* isolates analyzed (Figure 1). Isolates which share >90% genetic similarity as determined by the Dice coefficient are grouped by the same color in Figure 1. Several other genetic markers were included in Figure 1, including the translocations of supernumerary sequences to chromosome 3 [19]. In isolate 2516, two sequence blocks, each several 100 kb in size, translocated from the supernumerary chromosomes to core chromosome 3. Seven isolates in the collection are found to have the first insertion, and three of these also have the second insertion. This likely occurred in a sequential fashion: starting with a >204 kb translocation closest to the telomere of chromosome 3, followed by a 464 kb event further away from the telomere (883,738 bp). Figure 1 shows that an entire sub-cluster of the AFLP tree is made up of isolates containing the first insertion of supernumerary sequence.

### 2.2. Fusarium poae Likely Combines Sexual and Asexual Reproduction

It has been shown that RIP is associated with sexual reproduction [20]. Since RIP functions on the core genome of *F. poae* [19], it stands to reason that meiosis should be ongoing in this species. Moreover, the presence and conservation of the “meiotic toolbox” was shown [19]. We expanded these authors’ investigation into the distribution of MAT1-1 and MAT1-2 in *F. poae*. These results are visualized in Figure 1 and Table 1. The MAT1-1 idiomorph is predominantly present (81% MAT1-1, 19% MAT1-2; one undetermined, but both idiomorphs do co-occur in the same location (Table 1)). The presence of detectable recombination events dictates that *F. poae* should be a sexual species, even if the sexual stage has never been observed.

All *F. poae* isolates were screened for the presence of two major translocations from the supernumerary genome to chromosome 3 (see also [19]). Eight isolates containing the first translocation of supernumerary sequence (closest to the telomere of chromosome 3) were collected from different locations across Flanders, Belgium (Table 1). It can be assumed that this genotype has spread clonally from one location to the other two.

### 2.3. Transposable Element Proliferation between Near-Clonal Isolates

To uncover if TE insertions are shared between isolates, the location of TE insertions in isolates 2516 and 2531 were compared. According to Figure 1, these isolates are near-clonal (comparable to the AFLP pattern of the three repetitions of isolate 9125). Reads from an Illumina HiSeq run of DNAs from five different isolates, including isolate 2531 were mapped onto the reference assembly of isolate 2516 and 73 of the 135 TE copies on the core genome of 2516 did not have read support from the DNA read mix, including absence of coverage by reads originating from isolate 2531 (Table 2). These TEs represent copies uniquely present in isolate 2516, and they are absent in the same location in isolate 2531.

### 2.4. Assessing the Genetic Chemotype of F. poae Isolates

All three thrichothecene loci were positioned on the assembly of *F. poae* isolate 2516 within their exact genomic coordinates. The *Tri1* gene is located on chromosome 1 (55,207–56,959 bp). The organization of the *Tri1* region in isolate 2516 is identical to what was described previously by Proctor et al. [32]. The main trichothecene biosynthesis cluster (Tri5) is located on chromosome 2 (5,545,692–5,575,616 bp) and the *Tri101* gene can be found on chromosome 4 at (4,906,101–4,907,486 bp). These positions are similar to those for the three loci in *F. graminearum*.

To study the organization of the main trichothecene cluster, we applied the diagnostic PCR developed by Dinolfo et al. [33] to predict the genotype of all *F. poae* isolates towards NIV production. This assay is based on the *Tri7* gene. Using this approach, all isolates were genetically chemotyped to be of the NIV genotype.

**Figure 2:**
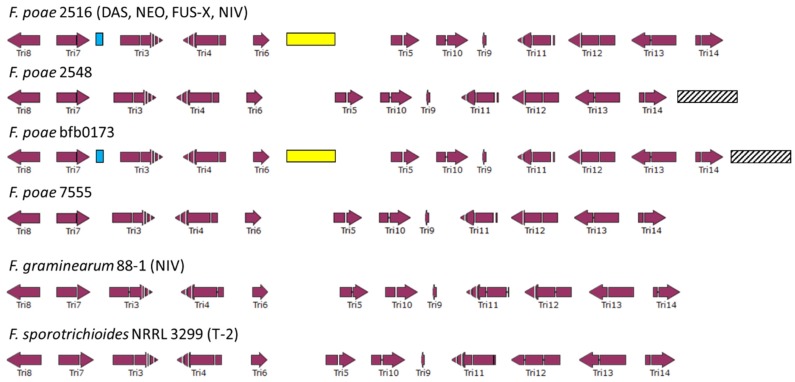
Organization of the main trichothecene biosynthesis cluster in *F. poae* (isolate 2516; NCBI accession LYXU01000002.1), which is identical to the organization in *F. graminearum* (isolate 88-1, NIV chemotype, NCBI accession AF336365) and *F. sporotrichioides* (isolate NRRL 3299, NCBI accession AF359360). The difference in Tri6-Tri5 intergenic region between *F. poae* and *F. graminearum/F. sporotrichioides* is explained by the presence of a RIPped pogo transposable element (yellow). This element is also present at the same location in isolate bfb0173. In isolates 2516 and bfb0173, there is a 400 bp repetitive element between Tri7 and Tri3 (blue). Finally, in isolates 2548 and bfb0173, there is a RIPped retrotransposon downstream of Tri14 (shaded box).

In a next step, the main trichothecene gene cluster was extracted from chromosome 2 of isolate 2516 (NCBI accession LYXU01000002.1). Figure 2 depicts the organization of the main cluster and the comparison with the gene clusters in *F. sporotrichioides* (isolate NRRL 3299, T-2 toxin chemotype, NCBI accession AF359360.3) and *F. graminearum* (isolate 88-1, NIV chemotype, NCBI accession AF336365.2). The overall organization of the main trichothecene biosynthesis cluster is highly similar to that previously described in *F. sporotrichioides* and *F. graminearum*. The largest difference is the intergenic distance between *Tri5* and *Tri6* that is expanded in isolate 2516. A low complexity (low GC %) region with similarity to a *pogo* TE accounts for an additional 2.1 kb of sequence in this region. The best Blastx hit for this element is “*pogo* transposable element with KRAB domain from *F. oxysporum* f. sp. *cubense* race 1” (NCBI accession ENH68388.1).

The genome sequences of isolates 2548, 7555 and bfb0173 were consulted to find differences in the organization of the main trichothecene biosynthesis cluster with isolate 2516 and these isolates. The main trichothecene biosynthesis cluster of isolates 2548, 7555 and bfb0173 can be found on contigs 77, 419 and 361 respectively. The RIPped *pogo* TE between *Tri5* and *Tri6* is also present in isolate bfb0173 and is highly conserved (three SNPs (single-nucleotide polymorphisms) between 2516 and bfb0173). It is not present in isolates 2548 and 7555. The *F. poae* collection was screened for the presence of this element (Figure S1). Thirty-eight isolates contain the *pogo* element at the same location as isolates 2516 and bfb0173.

Figure 1 visualizes the distribution of these isolates across the AFLP tree. Downstream of *Tri14* a RIPped retrotransposon occurs in isolates 2548 and bfb0173. In isolate bfb0173, it consists of 3.7 kb of low complexity sequence, while in isolate 2548 it is only partially assembled, with 1.2 kb of sequence that is almost identical to the sequence of isolate bfb0173 (10 SNPs). The best Blastx hit for this retrotransposon is a protein from *Claviceps purpurea* (NCBI accession CCE29311.1). In isolates 2516 and bfb0173, there is a 400 bp repetitive element without Blastx hits between *Tri7* and *Tri3* that is not present in isolates 2548 and 7555. The three insertions in and around the main trichothecene biosynthesis cluster (between *Tri7* and *Tri3*, between *Tri6* and *Tri5* and bordering *Tri14*) show a specific pattern, as different combinations of two insertions are not shared between the same isolates.

Finally, variation at the *Tri1* locus, on chromosome 1, is known to be responsible for chemotype diversity in *Fusarium* species [12,34]. The *Tri1* genes in isolates 2516 and bfb0173 are identical to one another and to the published sequence for *FpTri1* (NCBI accession GQ915520), and 97% sequence identity to the *Tri1* gene of isolates 2548 and 7555 (respectively 51 and 52 (=51 + 1) SNPs across the 1753 bp gene). Respectively 24 and 25 of these SNPs occur in the exons of the gene, while 87% of the gene is exonic (1527 bp). Remarkably, only two (out of 24) and three (out of 25) of these SNPs are non-synonymous mutations. Two of these amino acid substitutions occur at the N-terminal end of the protein within the first 14 amino acids. Figure S2 shows the read mapping of HiSeq reads from isolate bfb0173 and isolate 2548 on the published *FpTri1* type, and shows that a disproportionate amount of SNPs occurs in the introns of the genes.

**Figure 3:**
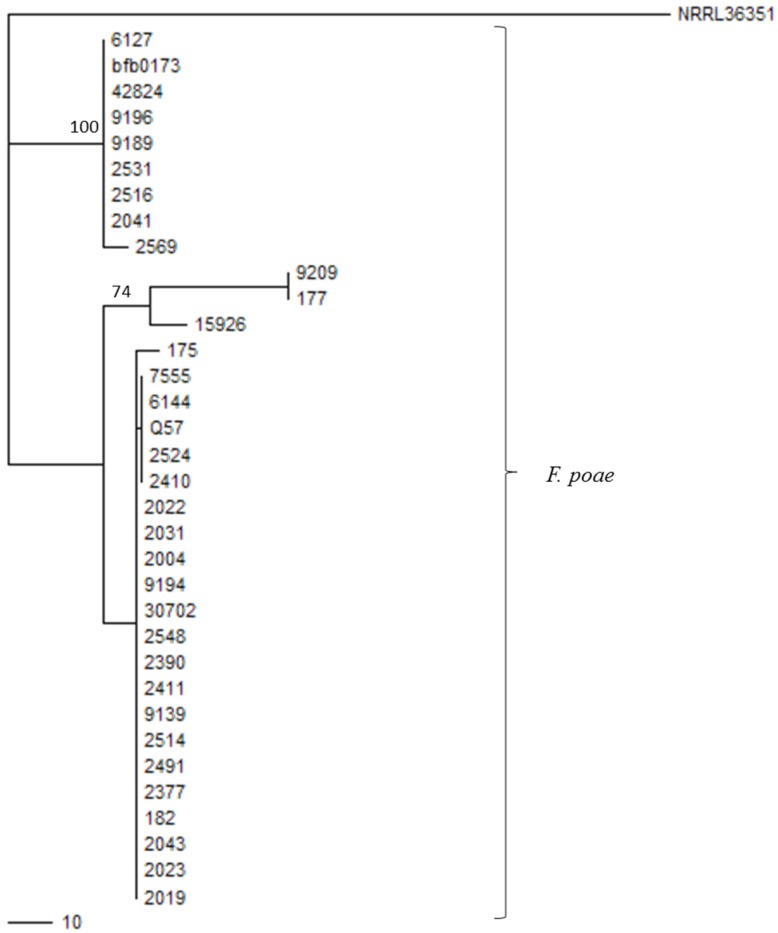
Maximum parsimony phylogenetic tree that was built from the 1100 bp fragments of the Tri1 gene with PAUP * 4.0b10. The tree is based on 74 parsimony informative characters. Bootstrap values were calculated with the PHYLIP package and those exceeding 70% are shown on the tree. *Fusarium* sp. NRRL 36351 (NCBI accession GQ915523.1) was used as outgroup, however, for construction of the phylogenetic tree, mid-point rooting was used.

A subset of 34 isolates of the *F. poae* collection was selected for sequencing of a 1100 bp fragment of *Tri1*. The existence of two major *Tri1* types within the collection was confirmed, with additional variation at the level of individual isolates (Figure 3).

### 2.5. Assessing the Phenotypic Chemotype of *F. poae* Isolates

**Figure 4:**
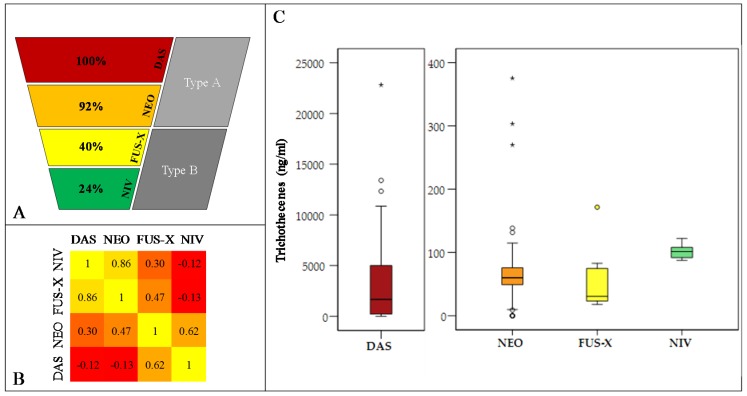
Summary of trichothecene biosynthesis in *F. poae* isolates. (**A**), simplified representation of the chemotype of DAS producing *F. poae* isolates. The chemotypes detected were strictly hierarchical, i.e., when compounds lower on the scheme are detected, the compounds higher on the scheme have been produced as well. (**B**), Pearson correlations between the four trichothecenes that are produced by *F. poae* isolates. (**C**), overview as Box-Whisker plots of the concentration ranges of the four trichothecenes. Note that the range of DAS is two orders of magnitude higher than the range for NEO, FUS-X and NIV concentrations. The boxes for every mycotoxin show the lower and upper quartile (delineating the boxes) of the measured concentrations (ng/mL) and the median (thick line within the boxes). The whiskers represent the minimum and maximum values. Circles and asterisks are outliers and extreme values which fall respectively outside of one-and-a-half additional box lengths and three additional box lengths counted from the upper quartile limit.

Figure 4 shows for 61 *F. poae* isolates the trichothecenes that were detected after growth in the trichothecene biosynthesis inducing medium. The values were log transformed as the amounts of DAS were higher than those of the other toxins. DAS is also the most common mycotoxin, with 59 of 61 isolates being positive for DAS, ranging from the LOD to 22804 ng/mL. Of these 59 isolates, 55 produced NEO in the range of the LOD to 375 ng/mL. Of this set, 24 isolates produced FUS-X in the range of the LOD to 172 ng/mL and, of these FUS-X producers, 14 isolates also produced NIV in the range of 88 ng/mL to 122 ng/mL. The hierarchical nature of these chemotypes is visualized in Figure 4A. Finally, there is no significant effect of the presence/absence of the *pogo* element on the chemotype, for any of the four detected trichothecenes (Mann–Whitney U test, *p* values > 0.05) (Figure 1 and Table 1).

Figure 4 summarizes the trichothecene production in vitro for all isolates. DAS production is about 50-fold higher compared to the production of NEO, FUS-X or NIV. Note that two isolates show outlier values for DAS and NEO production (isolate 30702 and isolate 9194). Concordantly, Figure 4B shows that production of DAS and NEO is correlated.

Table S1 shows the result of two biological repetitions of the chemotyping experiment for 28 isolates. Compounds with a plus sign were detected consistently, while compounds indicated with “X” were only detected in one of the two repetitions. For 20 of the isolates, the chemotype previously shown in Table 1, moved “further downstream”, with FUS-X or even NIV production where there was none in another iteration. This hierarchical nature of production was never violated in any of the strains in either of the repetitions. The chemotype of seven isolates was identical in the different repetitions: for example isolate 7555 only produced DAS, and isolate 2569 produced all four compounds.

## 3. Discussion

In this paper, we assessed genetic variation, sexual and asexual reproduction, the trichothecene clusters and the chemotype of a set of *F. poae* isolates. AFLP analysis on the *F. poae* isolates to assess genetic variation among isolates from geographic different areas was performed. The isolates show >75% similarity, suggesting that they are part of a single monophyletic lineage, which is similar to what was found before for *F. poae* [35,36]. No obvious connection was found between geography and the AFLP sub-clustering, which is in accordance with previous studies [21,25]. Still, isolate bfb0173 from China which is the most distant, clustered separately from all other isolates and the dominant genotype among the Italian isolates did not occur in Belgium. In accordance, Kerenyi, Taborhegyi, Pomazi and Hornok [22] reported a link between geographic origin and VCG/RAPD profile (Random amplification of polymorphic DNA). We subsequently researched whether there was a link between the position of isolates in the AFLP tree and the host plant. Five isolates from maize were included in the study, aside from isolates from wheat and barley. The maize isolates are scattered throughout the tree, suggesting that no significant host specialization has occurred within *F. poae*. This is in agreement with literature reports that suggest that most isolates have a broad host range [22]. However, a host preference cannot be excluded.

As species capable of combining both sexual and asexual reproduction, and species with an active arsenal of TEs, are considered to be the biggest challenges for durable disease management [16] and key trait of successful pathogens [29], we investigated both features in the *F. poae* isolates. A sexual cycle has never been shown for *F. poae*, but is highly likely to occur. However, the skewed mating type distribution encountered among the Belgian *F. poae* isolates indicates that meiosis does not occur at high frequency [19]. Although in oomycetes, such as *Phytophtora infestans*, an unbalanced distribution of the two mating types has been related to fitness costs, in *F. poae* there is no evidence for a similar link. Opposed to what was described recently by Dinolfo, Castanares and Stenglein [21], we did not find any evidence for homothallism in *F. poae*. To confirm the presence of a sexual cycle, RIP was studied in isolate 2516. This analysis showed that meiosis and RIP likely did not happen very recently, allowing the accumulation of intact TE on the core genome during asexual propagation [19]. The tight correlation between RIP and meiosis should offer opportunities for understanding the balance between clonal and sexual reproduction, by using TE insertions as markers for “track and trace” studies and monitoring when/if they become RIPped.

We showed that near-clonal isolates can differ heavily in TE proliferation, which may occur over very short evolutionary periods. It seems likely that such TE mobilization is obscuring the nearly clonal nature of some isolates. The occurrence of TE integrations during asexual reproduction, such as in in vitro cultures, may also account for the instability of single spore cultures reported previously for *F. poae* [22]. Moreover, novel genotypes that are generated through the presence of the supernumerary genome show large genetic divergence from other *F. poae* isolates when analyzed by a genome-wide method such as AFLP. These findings may explain the discrepancy in intra-species variability between genome-wide and multi-locus barcoding approaches that has been witnessed for *F. poae*. Genome-wide studies such as those employing RAPD [22], AFLP [21,23] and ISSR [21,25] consistently detect medium to high genetic diversity, which is in sharp contrast to multi-locus barcoding analyses [26,27]. The genome dynamics and the active TEs arsenal that are present in *F. poae*, may accelerate its evolution and lead to elevated levels of genetic diversity, without interfering with barcode diversity.

In *Fusarium* species within the *F. sambucinum* clade, trichothecene production is orchestrated by three loci comprising the Tri 5 gene cluster, the Tri1-Tri16 cluster and the Tri 101 gene. Within the trichothecene type B producing FGSC (*Fusarium graminearum* species complex), it is common to define the chemotype of an isolate by its genotype, determined by a diagnostic PCR of a differential trichothecene biosynthesis gene [37]. A similar method has been developed for the NIV chemotype in *F. poae* using the *Tri7* gene [33]. All isolates in the study of Dinolfo et al. [21], all isolates in the study by Covarelli et al. [7], and all isolates in our study were genetically determined to have the NIV chemotype. Another important locus determining trichothecene production is *Tri1*. Variations in *Tri1* sequence are responsible for recent shifts in trichothecene production within *F. graminearum* this species [34]. Remarkably in *F. poae*, different haplotypes of the *FpTri1* gene can also be found although most SNPs are synonymous mutations. The existence of two major *Tri1* types within the collection was confirmed, with additional variation at the level of individual isolates. As both types code for highly similar enzymes, this variability cannot explain the variable phenotypic chemotype. Finally, we studied the *Tri5* gene cluster in *F. poae*. In *F. poae* isolate 2516, this main trichothecene cluster and neighboring genes are similar in organization to the trichothecene clusters in *F. graminearum* and *F. sporotrichioides* [38]. However, uniquely for a *Fusarium* species, but in line with the unique genome dynamics of *F. poae*, the main trichothecene biosynthesis cluster and its environment are invaded by TEs. The insertion of repetitive sequences into the genome is a useful tool for track and trace studies in fungal populations, as every such insertion can be considered a unique event. In fact, all sequenced genome contain a unique pattern of TE insertions in the trichothecene biosynthetic gene cluster.

Using the three insertions across the trichothecene biosynthesis cluster, a recombination event was uncovered illustrating the presence of a sexual cycle in *F. poae*. In *F. graminearum*, it was shown that the main trichothecene cluster, with similar genomic coordinates as in *F. poae*, is situated in a region of the genome with an elevated recombination rate [39]. Ward et al. [40] have shown that the composition of the main trichothecene cluster in type B producing species is the result of multiple recombination events. O’Donnell et al. [41] have shown that the main trichothecene biosynthesis cluster in *F. aethiopicum* (a member of the FGSC) is the result of inter-chemotype recombination. That cluster is the result of recombination between different haplotypes in these species independently, but it remains to be seen what the functional consequences for trichothecene production have been. The localization of a RIPped *pogo* element between *Tri6* and *Tri5* in isolates 2516 and bfb0173 is remarkable. No intact copies of this element could be found in the core genome, and it was likely RIPped to extinction in isolate 2516 [19].

In the *F. equiseti* complex, this space is occupied by an additional Zn2Cys6 transcription factor [32]. There is a strong link between transposable elements and transcription factors (TF). Many TF-associated DNA binding sites are derived from ancient transposable elements and TEs have played a defining role in the formation of complex regulatory networks [42]. Moreover, some TF families are derived from ancient TE domestication [43]. There is no similarity between the *pogo* element in *F. poae* and the Zn2Cys6 TF in *F. equiseti* (data not shown), so an evolutionary link between the two seems unlikely. Nevertheless, insertions into the *Tri6-Tri5* intergenic region have been shown to influence trichothecene production [44].

To investigate whether the variability in trichothecene genes influenced the production of trichothecenes (i.e., the phenotypic chemotype), we monitored the production of both type A (DAS and NEO) and type B (FUS-X and NIV) trichothecenes. Remarkably, there was no correlation between any of the variations at the level of the *Tri* genes (Tri1, *pogo* element between *Tri5* and *Tri6*) and the production of type A and Type B trichothecenes.

Other researchers addressed this phenotypic chemotype of *F. poae*. Pasquali et al. [45] used agmatine rather than L-arginine as a nitrogen source in their trichothecene biosynthesis inducing medium and detected FUS-X and NIV production from four *F. poae* isolates, with large inter-isolate differences. Previous studies have utilized autoclaved cereal substrates [15,23] and in planta inoculations [46]. The discrepancies in chemotype depending on the matrix or medium (and nitrogen source) used, may lead to an underestimation of certain toxicological aspects of *F. poae*. The Italian isolates in our study were previously chemotyped by growth on autoclaved wheat. Three that were at that time found to produce no type A trichothecenes, produced both DAS and NEO in the mineral medium. This is an important point to make, as, at least in certain assays, type A trichothecenes are more toxic than type B trichothecenes [47,48].

Despite all the variability in trichothecene production between isolates, the chemotype was shown to be strictly hierarchical in all isolates, implying that when NIV is detected in *F. poae*, Type A trichothecenes are also synthesized. Inoculations in cereal matrices seemingly shift the balance more towards type B production (NIV), while the in vitro mineral medium with l-arginine mainly stimulated DAS production [46]. However, trichothecenes of the DAS-related group such as mono-acetyl scirpenol and scirpenol are often overlooked in mycotoxin surveys, while they may be important compounds in nature, particularly associated with *F. poae* [49,50].

Finally, although *F. poae* is often reported as T-2 toxin and HT-2 toxin producing species, our data on the TRI genes detected in *F. poae* confirmed previous reports on the lack of the key gene related to the production of both mycotoxins in the trichothecene cluster [32]. This achievement is extremely important for a correct risk assessment of the *F. poae* occurrence on agro-food important crops, since T-2 toxin and HT-2 toxin are the most acute poisoning mycotoxins produced by Fusarium species and both are under evaluation at European level for their possible limits in food products.

## 4. Conclusions

Using a combined approach of AFLP and MAT locus analysis, we identified hallmarks of both sexual recombination and clonal spread of isolates in the population. Based on Tri7 sequence analysis, all *F. poae* isolates had the NIV chemotype, although not all isolates produced NIV in vivo. *Tri* gene clusters showed two layers of genetic variability. Firstly, the Tri1 locus was highly variable with mostly synonymous mutations and mutations in introns. Secondly, in a subset of isolates, the main trichothecene gene cluster was invaded by a transposable element between *Tri5* and *Tri6*. Nevertheless, these sources of variability could not explain variations in the phenotypic chemotype.

## 5. Materials and Methods

### 5.1. *Fusarium* Collection

For the purpose of this study, a broad collection of *Fusarium* isolates was gathered (Table 1). Of the 69 *F. poae* isolates, forty-one isolates were collected from fields in Flanders, Belgium. Ten Italian isolates were previously described [23]. Four isolates (Norwegian isolates) were donated by Dr. Anne van Diepeningen. One isolate (PD93/1780) originated from the *Fusarium* collection at Wageningen university (WUR). Twelve isolates came from the MUCL culture collection (MUCL, Louvain-La-Neuve, Belgium) and originated from maize and wheat. One Chinese isolate was included in the study (bfb0173).

Eight *F. graminearum* isolates and five *F. culmorum* isolates were obtained from Belgian fields as described above and the *F. graminearum* 8/1 isolate was kindly provided by Dr. Karl-Heinz Kogel. The *F. sporotrichioides* isolate (MUCL6133) and *F. langsethiae* isolate (MUCL34988) were purchased from the MUCL collection. One *F. langsethiae* isolate (CBS11324) was purchased from the CBS collection (Utrecht, The Netherlands). The other *F. langsethiae* isolates were kindly donated by Dr. Simon Edwards (Harper Adams College, UK) and Dr. Ingerd Hofgaard (Bioforsk, Norway) respectively. For a detailed overview of these isolates, we refer to Table S3.

Field samples, presumed to consist of a mix of species and genotypes, were obtained and confirmed to contain *F. poae* as described in Audenaert, Van Broeck, Bekaert, De Witte, Heremans, Messens, Hofte and Haesaert [5]. These were purified to a single spore level according to a method by Dr. Susanne Vogelgsang (Agroscope, Zürich, Switzerland, personal communication). Mycelium plugs were placed on a PDA (potato dextrose agar) plate for 7 days under a light regime of UV/darkness for sporulation (12 h (365 nm 10 W)/12 h). Sporulated mycelium was resuspended in 9 mL of distilled water. After vortexing and diluting, the resulting conidial dilutions were poured on plates containing water agar. Conidia were allowed to settle briefly after which the suspension was poured off. Plates were incubated for 16 h in the dark in a slanted position. Finally, typical *F. poae* spores were isolated using a Pasteur pipette.

DNA extraction of isolates was performed as described by Audenaert et al. [5]. Isolates confirmed to be *F. poae* with primers Fp82F/R (Table S2) [51] were preserved at −80 °C as spores in a 20% glycerol solution.

### 5.2. Phylogenetic Analyses

DNA for AFLP analyses was extracted with the Invisorb Spin Plant MiniKit (Invitek, Berlin, Germany) according to the manufacturer’s instructions. DNAs were quantified with the Nanodrop 1000 system (ThermoFisher Scientific, Merelbeke, Belgium) and AFLP analysis was performed as described by Vos et al. [24]. For selective amplification, four primer pairs, EcoRI-AC/MseI-CC, EcoRI-AC/MseI-CA, EcoRI-AC/MseI-CG and EcoRI-GG/MseI-CA were used. Fragments were analyzed on a CEQ 2000 Genetic Analysis System (Beckman Coulter, Fullerton, California) and visualized with the Genographer 1.6.0 software (Benham, Montana State University, Bozeman, Montana). Bands were scored visually and data was processed as described in Scauflaire et al. [52]. From the visual scoring, a binary matrix was constructed and UPGMA cluster analysis was performed based on the Dice similarity coefficients between the isolates. To simulate the AFLP pattern of a clonal isolate, and to determine the genotyping error rate, three DNA subsamples from one isolate were included in the experiment.

### 5.3. *Fusarium poae* Genome Data

The sequence of the trichothecene biosynthesis loci in isolates bfb0173, 2548 and 7555 was extracted from their assemblies and compared with the genome sequence of isolate 2516 (NCBI accession LYXU00000000.1 [19]). The annotation of isolate 2516 was checked to ascertain that genes were correctly annotated, in accordance with literature on related *Fusarium* species. Similarities between amino acid sequence of the predicted proteins, and between nucleotide sequence of the intergenic regions, were determined with ClustalW2 [53].

To visualize differences between the different Tri1 types, the HiSeq reads of isolates bfb0173 and 2548 were mapped to the only *Tri1* type already published (NCBI accession GQ915520) with CLC Genomics Workbench 7.5. Reads were mapped at 0.5 length fraction and 0.8 similarity fraction.

DNA from five *F. poae* isolates (6127, 6114, 42824, 30702 and 2531) was pooled in a 1:1:1:1:1 ratio. This DNA mix was sequenced with Illumina technology as outlined by Vanheule et al. (2016) [19]. The resulting HiSeq reads cannot be separated and are therefore a hybrid pool of five isolates. Investigation into identical integration of TEs was performed as outlined by Vanheule et al. [19].

### 5.4. Trichothecene Production Analyses

A trichothecene biosynthesis inducing medium described by Gardiner et al. [54] was used. The general composition of the medium was identical as described in their study, but Phytagel was excluded and NaNO_3_ was exchanged with l-arginine (Duchefa Biochemie, Haarlem, The Netherlands) at 5 mM as Gardiner et al. [54] showed that this nitrogen source induces trichothecene biosynthesis in *F. graminearum*. Medium was prepared in double concentration. Conidia were harvested by adding distilled water amended with 0.01% Tween80 (Merck, Darmstadt, Germany) to the fully grown PDA plates and by rubbing the mycelium with a spatula. Conidia were counted with a Bürker counting chamber and diluted to a final concentration of 2 × 10^6^ conidia/mL. Finally, 0.5 mL of double concentrated medium and 0.5 mL of double concentrated conidial suspension were added together in 24 well plates (leading to 1× concentration of medium and 1× concentration of conidia). The 24 well plates were closed with a lid and the fungus grew stationary under 16 h light/8 h dark regime at 22 °C. The medium and fungal mass was removed from the wells. After seven days, supernatant was extracted from the cultures by centrifugation at 2750 g for 10 min and immediately processed for LC-MS/MS analysis.

Sample preparation and LC-MS/MS followed an “evap and shoot” principle, without extensive sample cleanup. Individual mycotoxin solid standards (1 mg) of DON, NIV, NEO, FUS-X, T-2 toxin, HT-2 toxin, DAS and deepoxydeoxynivalenol (DOM) were supplied by Coring System Diagnostics (Gernsheim, Germany) as certified solutions. All mycotoxin solid standards were dissolved in acetonitrile (1 mg/mL), and were storable for a minimum of 1 year at −18 °C [55]. Working solutions of 10 ng/μL for DON, NIV, NEO, FUS-X, T-2 toxin, HT-2 toxin, DAS and DOM were prepared in methanol and stored at −18 °C. From the individual working solutions, a mixture was prepared in methanol, stored at −18 °C and renewed monthly with the following concentrations: the mycotoxin mix (mycotoxins, 10 ng/μL) and the internal standard (DOM, 10 ng/μL).

An aliquot of the trichothecene biosynthesis inducing medium was transferred in a 10 mL glass tube and the internal standard was added (DOM, 10 μL, final concentration 100 ng/mL). To construct a calibration curve, five blank aliquots were spiked with the mycotoxin mix in an increasing concentration range (2 μL (20 ng/mL), 5 μL (50 ng/mL), 10 μL (100 ng/mL), 15 μL (150 ng/mL) and 20 μL (200 ng/mL), respectively). Samples were vortexed for 2 min (Labinco, Breda, The Netherlands) and spiked medium was evaporated to dryness under a gentle N_2_-stream at 60 °C using the Turbovap^®^ LV Evaporator (Biotage, Uppsala, Sweden). The dried residue was redissolved in 100 μL injection solvent, which consisted of 70% mobile phase A (water/methanol (95/5, *v*/*v*) + 5 mM ammonium acetate, 0.1% glacial acetic acid) and 30% mobile phase B (water/methanol (95/5, *v*/*v*) + 5 mM ammonium acetate, 0.1% glacial acetic acid). Prior to injection, the sample was vigorously vortexed for 2 min, collected in a 0.22 μm Ultrafree-MC centrifugal device (Millipore, Bedford, MA, USA) and centrifuged for 10 min at 10,000 *g*. LC-MS/MS methodology was as detailed in Vanheule, Audenaert, De Boevre, Landschoot, Bekaert, Munaut, Eeckhout, Hofte, De Saeger and Haesaert [8]. LODs and LOQs for the trichothecenes produced by *F. poae* were, respectively, 30 and 61 ng/mL (NIV), 31 and 63 ng/mL (FUS-X), 39 and 78 ng/mL (NEO) and 41 and 82 ng/mL (DAS). Certain samples showed peaks which according to the calibration curve corresponded to concentrations between the LOD and the LOQ for DAS, NEO and FUS-X. These samples were assigned the LOD concentration as they could not be reliable quantified.

Samples with concentrations out of the range of the calibration curve were diluted and reanalyzed. To circumvent the possible matrix effect issue, a new calibration curve was constructed with a blank sample. This blank sample was also diluted, using the same protocol as the unknown diluted samples. Internal standards and spiked concentrations were then added anew.

Data acquisition and processing was performed with MassLynx 4.1 and QuanLynx 4.1 software respectively (Micromass, Manchester, UK). Pearson correlations between the levels of trichothecenes were determined with Microsoft Excel 2013. The concentration range of the trichothecenes was visualized in Box-Whiskers plots, constructed with SPSS22 (IBM, Armonk, NY, USA). The effect of the *pogo* insertion on trichothecene production was analyzed with the Mann–Whitney U test in SPSS.

### 5.5. Diagnostic PCRs and Amplicon Sequencing

All primers are listed in Table S2. Mating type was determined as described by Kerenyi et al. (2004). For the two insertions of supernumerary sequence into the core chromosomes (INS1 and INS2), primers were used as described in Vanheule et al. (2016). A 1100 bp fragment of the *Tri1* gene was amplified as described in Proctor et al. (2009) for a set of 34 *F. poae* isolates selected from different backgrounds and chemotypes. The presence of a *pogo* TE in the Tri cluster was investigated with three primers as detailed in Table S2. PCR reactions were done with Promega GoTaq G2 Polymerase according to the manufacturer’s instructions (Promega, Leiden, The Netherlands). Annealing temperatures were chosen, based on Promega’s Biomath Calculator. PCRs were performed in an Applied Biosystems GeneAmp PCR System 9700, amplicons were separated on 1.5% (*w*/*v*) agarose gels stained with 0.1 μg/mL ethidium bromide, and visualized with a Biorad Gel Doc XR+.

PCR products were purified with the EZNA Cycle-Pure kit (VWR Chemicals, Haasrode, Belgium) and sequenced in both directions by Macrogen Inc. (Amsterdam, The Netherlands). Forward and reverse sequences were curated with CLC Main Workbench 7. Maximum parsimony analysis was performed with PAUP version 4.0b10 [56]. The *Tri1* fragment from *Fusarium sp.* NRRL 36351 (NCBI accession GQ915523.1) was used as the outgroup, however for construction of the phylogenetic tree mid-point rooting was used. Bootstrapping was performed with PHYLIP based on 1000 replicates [57].

## Figures and Tables

**Table 1:** List of *F. poae* isolates (*n* = 69) used in this study. Location, host, year of isolation and mating type are shown. The ID is the identification number in our local collection. Concentrations of mycotoxins are expressed as ng/mL. ND: not detectable (<LOD). >LOD: amount above the LOD but below the LOQ. NCA: no chemotype available (not determined). DAS: diacetoxyscirpenol; NEO: Neosolaniol; FUS-X: Fusarenon-X; NIV: Nivalenol. Absence (-) or presence (+) of two supernumerary sequence insertions in chromosome 3 (INS1: >204 kb and INS2: 464 kb) are indicated in the table. Availability of an AFLP profile is indicated by “+” or “-“.

ID	Location	Host	Year	Reference	Mating Type	INS1	INS2	AFLP	DAS	NEO	FUS-X	NIV
175	Aas, Norway	barley	1996	Sundheim L. unpubl.	MAT1-2	-	-	-	355	55.1	ND	ND
177	Norway	wheat	1996	Sundheim L. unpubl.	MAT1-1	-	-	+	NCA			
182	Norway	barley	1996	Sundheim L. unpubl.	MAT1-1	-	-	+	NCA			
185	Norway	barley	1996	Sundheim L. unpubl.	MAT1-1	-	-	+	ND	ND	ND	ND
1879	Bottelare, Belgium	wheat	2010	this study	MAT1-1	-	-	+	2160	66	25	88
2004	Zwevegem, Belgium	wheat	2010	this study	MAT1-1	-	-	+	89	ND	ND	ND
2019	Zwevegem, Belgium	wheat	2010	this study	MAT1-1	-	-	+	231	54	ND	ND
2022	Zwevegem, Belgium	wheat	2010	this study	MAT1-1	-	-	+	1588	62	18	ND
2023	Zwevegem, Belgium	wheat	2010	this study	MAT1-1	-	-	+	44	12	ND	ND
2028	Zwevegem, Belgium	wheat	2010	this study	MAT1-1	-	-	+	3650	79	77	93
2031	Zwevegem, Belgium	wheat	2010	this study	MAT1-1	-	-	+	109	ND	ND	ND
2033	Zwevegem, Belgium	wheat	2010	this study	MAT1-1	-	-	+	1306	59	ND	ND
2041	Zwevegem, Belgium	wheat	2010	this study	MAT1-2	-	-	+	60	12	>LOD	108
2043	Zwevegem, Belgium	wheat	2010	this study	MAT1-1	-	-	+	3971	72	ND	ND
2044	Zwevegem, Belgium	wheat	2010	this study	MAT1-2	-	-	+	144	9	ND	ND
2056	Zwevegem, Belgium	wheat	2010	this study	MAT1-1	-	-	+	NCA			
2371	Bottelare, Belgium	wheat	2011	this study	MAT1-2	-	-	+	6923	101	ND	ND
2375	Bottelare, Belgium	wheat	2011	this study	MAT1-1	-	-	+	15	10	ND	ND
2377	Bottelare, Belgium	wheat	2011	this study	MAT1-1	-	-	+	31	11	>LOD	103
2380	Bottelare, Belgium	wheat	2011	this study	MAT1-1	-	-	+	1743	64	ND	ND
2381	Bottelare, Belgium	wheat	2011	this study	MAT1-1	-	-	+	222	54	ND	ND
2390	Bottelare, Belgium	wheat	2011	this study	MAT1-1	-	-	+	3027	67	ND	ND
2392	Bottelare, Belgium	wheat	2011	this study	MAT1-1	-	-	+	196	14	ND	ND
2395	Bottelare, Belgium	wheat	2011	this study	MAT1-1	-	-	+	193	55	ND	ND
2410	Bottelare, Belgium	wheat	2011	this study	MAT1-1	-	-	+	9562	84	ND	ND
2411	Bottelare, Belgium	wheat	2011	this study	MAT1-1	-	-	+	238	54	ND	ND
2424	Koksijde, Belgium	wheat	2011	this study	MAT1-1	-	-	+	2098	59	ND	ND
2476	Poperinge, Belgium	wheat	2011	this study	MAT1-1	-	-	+	488	75	172	103
2491	Poperinge, Belgium	wheat	2011	this study	MAT1-2	-	-	+	12,336	99	ND	ND
2514	Zwevegem, Belgium	wheat	2011	this study	MAT1-1	-	-	+	2800	73	83	116
2516	Zwevegem, Belgium	wheat	2011	this study	MAT1-1	+	+	+	1126	57	ND	ND
2517	Zwevegem, Belgium	wheat	2011	this study	MAT1-1	-	-	+	641	58	ND	ND
2519	Zwevegem, Belgium	wheat	2011	this study	MAT1-2	-	-	+	9766	139	ND	ND
2521	Zwevegem, Belgium	wheat	2011	this study	MAT1-1	+	-	-	ND	ND	ND	ND
2524	Zwevegem, Belgium	wheat	2011	this study	MAT1-1	-	-	+	380	17	ND	ND
2525	Zwevegem, Belgium	wheat	2011	this study	MAT1-1	-	-	+	2195	61	ND	ND
2531	Zwevegem, Belgium	wheat	2011	this study	MAT1-1	+	+	+	803	59	22	92
2532	Zwevegem, Belgium	wheat	2011	this study	MAT1-1	-	-	+	1940	66	25	ND
2547	Zwevegem, Belgium	wheat	2011	this study	no amplicon	-	-	+	NCA			
2548	Zwevegem, Belgium	wheat	2011	this study	MAT1-1	-	-	+	67	ND	ND	ND
2565	Zuienkerke, Belgium	wheat	2011	this study	MAT1-1	+	-	+	69	18	>LOD	115
2569	Zuienkerke, Belgium	wheat	2011	this study	MAT1-1	+	-	+	4910	44	>LOD	122
2570	Zuienkerke, Belgium	wheat	2011	this study	MAT1-1	+	-	+	6630	66	25	ND
2571	Zuienkerke, Belgium	wheat	2011	this study	MAT1-1	+	-	+	NCA			
2671	Linter, Belgium	wheat	2011	this study	MAT1-1	+	+	+	2457.8	75	37	100
6127	Wageningen, The Netherlands	wheat	1964	MUCL	MAT1-2	-	-	-	10,864	303	75	ND
6114	Denmark	barley	1964	MUCL, Hennebert G.L. unpubl.	MAT1-2	-	-	+	135	55	ND	ND
7555	Heverlee, Belgium	wheat	1965	MUCL	MAT1-1	-	-	-	1067.8	ND	ND	ND
9125	Ferrara, Italy	wheat	2005	Somma et al. (2010)	MAT1-1	-	-	+	5096	73	ND	ND
9139	Ferrara, Italy	wheat	2005	Somma et al. (2010)	MAT1-2	-	-	+	8	ND	ND	ND
9181	Ferrara, Italy	wheat	2005	Somma et al. (2010)	MAT1-2	-	-	+	NCA			
9186	Ferrara, Italy	wheat	2005	Somma et al. (2010)	MAT1-1	-	-	+	8024	77	24	ND
9189	Ferrara, Italy	wheat	2005	Somma et al. (2010)	MAT1-1	-	-	+	6342	98	ND	ND
9192	Ferrara, Italy	wheat	2005	Somma et al. (2010)	MAT1-1	-	-	+	10,415	132	46	91
9194	Ferrara, Italy	wheat	2005	Somma et al. (2010)	MAT1-1	-	-	+	13,404	270	81	ND
9196	Ferrara, Italy	wheat	2005	Somma et al. (2010)	MAT1-2	-	-	+	3610	115	34	ND
9203	Ferrara, Italy	wheat	2005	Somma et al. (2010)	MAT1-1	-	-	+	115	55	ND	ND
9209	Ferrara, Italy	wheat	2005	Somma et al. (2010)	MAT1-1	-	-	+	5502	115	28	ND
11456	Heverlee, Belgium	barley	1968	MUCL, Meyer J.A. unpubl.	MAT1-2	-	-	+	264	20	>LOD	103
30702	unknown	in vitro plant	1990	MUCL, Marchand D. unpubl.	MAT1-2	-	-	+	22,804	375	57	ND
15926	Quebec, Canada	wheat	1970	MUCL, Hennebert G.L. unpubl.	MAT1-1	-	-	+	NCA			
42824	Belgium	wheat	2000	MUCL	MAT1-1	-	-	-	2120	73	ND	ND
bfb0173	China	barley	2005	Yang et al. (2008)	MAT1-1	-	-	+	NCA			
F49	Ath, Belgium	maize	2007	MUCL (Scaufflaire J.)	MAT1-1	-	-	+	5720	76	ND	ND
K46	Ath, Belgium	maize	2007	MUCL (Scaufflaire J.)	MAT1-1	-	-	+	5339	100	ND	ND
L24	Buissenal, Belgium	maize	2007	MUCL (Scaufflaire J.)	MAT1-1	-	-	+	162	55	ND	ND
Q57	Buissenal, Belgium	maize	2007	MUCL (Scaufflaire J.)	MAT1-1	-	-	+	916	56	ND	ND
S46	Villeroux, Belgium	maize	2007	MUCL (Scaufflaire J.)	MAT1-1	-	-	+	1208	59	ND	ND
PD93/1780	The Netherlands	carnation	2003	WUR, Waalwijk et al. (2003)	MAT1-1	-	-	+	4008	66	23	ND
*n* = 69					1 not determined 13 MAT1-2 55 MAT1-1	8 isolates containing INS1	3 isolates containing INS2	AFLP for 64 isolates	chemotype for 61 isolates; no chemotype analysed for 8 isolates			

**Table 2:** Transposable elements at unique locations in isolate 2516. List of TEs found on the core genome of isolate 2516, as well as those that do not have any read support from reads of a DNA mix consisting of five isolates, including isolate 2531. These are TE copies that are not present in the same location in isolate 2531, and this is a lower bound as there is no way to discern in which isolate(s) the remaining TEs are present (reads cannot be traced back to isolates). #, indicates the number of copies.

			# On the Core Genome of 2516	# Present in 2516 but Not in 2531 (No Read Support)
Superfamily		Family		
Retrotransposons				
	RLG_*Maggy*	Gypsy/Ty3 like	27	1
	RLG_*Skippy*	Gypsy/Ty3 like	5	2
DNA transposons				
	DTF_*Fot4*	Pogo	1	0
	DTF_*Fot5-A*	Pogo	40	26
	DTF_*ESP4-B*	Pogo	12	0
	DTF_*Drogon*	Pogo	33	33
	DTF_*Viserion*	Pogo	8	3
	DTM_*Hop7*	Mutator	8	8
	DTM_*Hop4*	Mutator	1	0
Sum			135	73

## Supplementary Materials

The following are available online at www.mdpi.com/2072-6651/9/9/255/s1, Figure S1: Diagnostic PCR of the pogo insertion between Tri5 and Tri6, Figure S2: Read mapping of reads from isolate bfb0173 (A and C) and isolate 2548 (B and D) on NCBI accession GQ915520, which is the only sequence of *FpTri1* available [32], Table S1: Chemotypes of 28 *F. poae* isolates in two biological repeats, Table S2: List of all primers used in this study, Table S3: Isolates from additional Fusarium species collected for this study.
