# Supplementary material for: Genetic Divergence and Chemotype Diversity in the Fusarium Head Blight Pathogen Fusarium poae
Source: Toxins (Basel). 2017 Aug 23;9(9):255. doi: 10.3390/toxins9090255 (PMC5618188; doi:10.3390/toxins9090255)
Supplement: Supplementary file 1 [file toxins-09-00255-s001.pdf]

Supplementary Materials: Genetic Divergence and Chemotype Diversity in the Fusarium Head Blight Pathogen *Fusarium poae*

Adriaan Vanheule, Marthe De Boevre, Antonio Moretti, Jonathan Scauflaire, Françoise Munaut, Sarah De Saeger , Boris Bekaert, Geert Haesaert, Cees Waalwijk, Theo van der Lee and Kris Audenaert

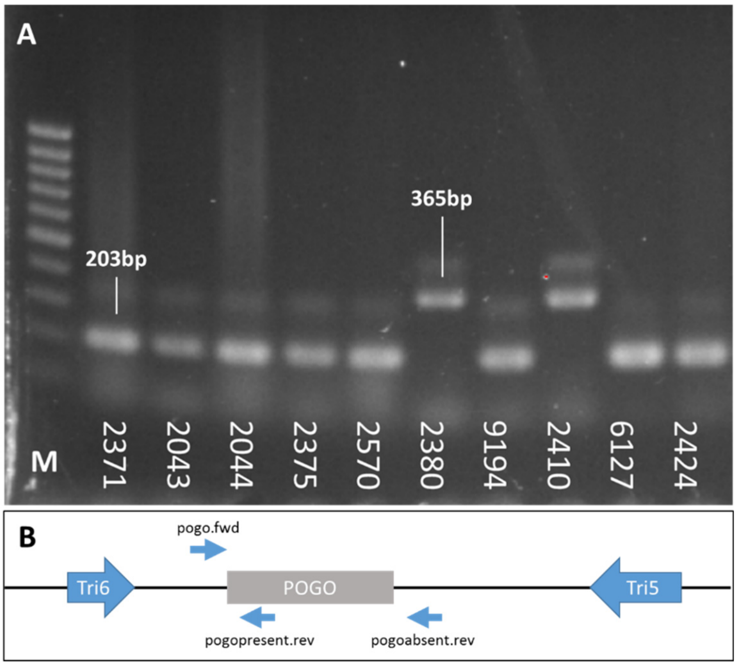

Figure S1. Diagnostic PCR of the pogo insertion between Tri5 and Tri6. **A**: Gel electrophoresis of the diagnostic PCR. **B**: Primers pogo.fwd and pogopresent.rev result in an amplicon of 203 bp (isolates 2371, 2043, 2044, 2375, 2570, 9194, 6127 and 2524), primers pogo.fwd and pogoabsent.rev (Table S2.) result in an amplicon of 365 bp (isolates 2380 and 2410). M represents the molecular weight marker (Thermofisher's Massruler DNA Ladder Mix, Low range).

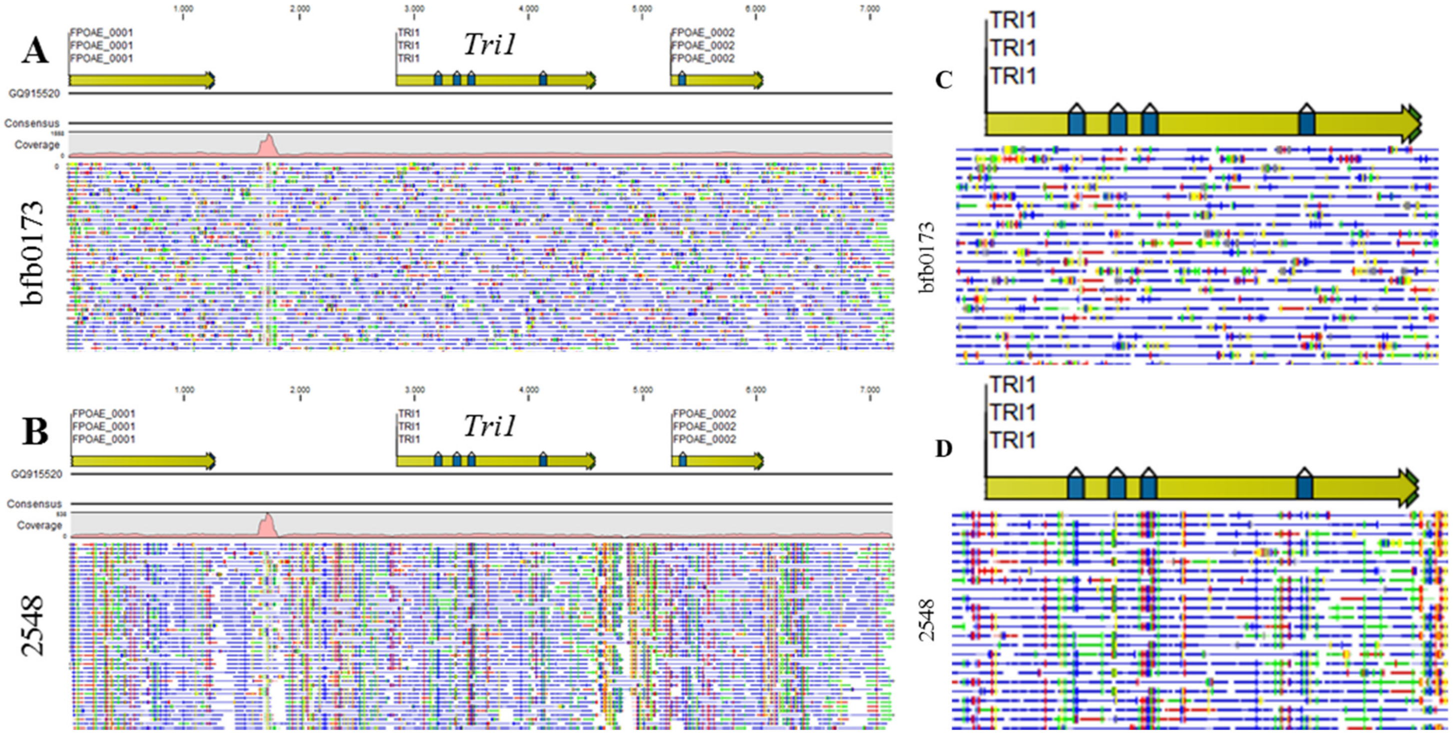

Figure S2. Read mapping of reads from isolate bfb0173 (**A and C**) and isolate 2548 (**B and D**) on NCBI accession GQ915520, which is the only sequence of *FpTri1* available [32]. The published allele is identical to the one in isolate 2516 and bfb0173. Paired reads are mapped as blue and they do not show any consistent SNPs (Single nucleotide polymorphisms, which are designated with divergent colors). A second *FpTri1* type identified in our study, occurred in isolates 2548 and 7555. Most SNPs are located in introns (e.g. intron 3) or in intergenic regions, and SNPs in the exons are predominantly synonymous by far.

Table S1. Chemotypes of 28 *F. poae* isolates in two biological repeats. "+" denotes trichothecenes which were consistently detected. "(+)" represents trichothecenes which were detected in one of the repeats. "-" means this trichothecenes were not detected in either of the repeats. DAS: diacetocyscirpenol, NEO: neosolaniol, FUS-X: fusarenon-X, NIV: nivalenol.

Isolate	DAS	NEO	FUS-X	NIV
2371	+	+	(+)	-
2375	+	+	(+)	-
2377	+	+	+	(+)
2381	+	+	(+)	-
2390	+	+	(+)	(+)
2392	+	+	(+)	-
2395	+	+	(+)	-
2411	+	+	-	-
2491	+	+	(+)	-
2514	+	+	+	(+)
2516	+	+	(+)	(+)
2517	+	+	(+)	-
2519	+	+	(+)	-
2521	(+)	(+)	-	-
2524	+	+	(+)	-
2525	+	+	(+)	-
2531	+	+	(+)	(+)
2532	+	+	+	-
2548	+	(+)	(+)	-
2569	+	+	+	+
S46	+	+	-	-
F49	+	+	(+)	-
6114	+	+	-	-
6127	+	+	+	-
7555	+	-	-	-
11456	+	+	+	(+)
30702	+	+	+	(+)
42824	+	+	(+)	-

Table S2. List of all primers used in this study.

ID	Sequence (5' → 3')	Reference	Target
POA-1-F	GCCTCACACTTTTTTCCTTCTTC	Kerenyi et al. (2004)	MAT1-1
POA-1-R	CAGTAAACCGGAATCATCAACG	Kerenyi et al. (2004)	MAT1-1
POA-2-F	ACGTACCATCTGACACTTGCTCG	Kerenyi et al. (2004)	MAT1-2
POA-2-R	AGTCGAGGAGGTCGTCAATCAAT	Kerenyi et al. (2004)	MAT1-2
Fp82F	CAAGCAAACAGGCTCTTCACC	Parry and Nicholson (1996)	EF-1α
Fp82R	TGTTCCACCTCAGTGACAGGTT	Parry and Nicholson (1996)	EF-1α

INS1-FLANK-fwd	CAGCGACTTGGTTCCGTATG	Vanheule et al. (2016)	Insertion 1
INS1-BLOCK-rev	GAAGCTTGTGACCACCCAAG	Vanheule et al. (2016)	Insertion 1
INS1-BLOCK-fwd	AGGTTCCGTCTTACTGGGTG	Vanheule et al. (2016)	Insertion 1
INS1-FLANK-rev	TCAACCAAGGCGTCGAAAAG	Vanheule et al. (2016)	Insertion 1
INS2-FLANK-fwd	GCATTGTGACGGATGGTACC	Vanheule et al. (2016)	Insertion 2
INS2-BLOCK-rev	GGTCTCACGATTTTCAGGCG	Vanheule et al. (2016)	Insertion 2
INS2-BLOCK-fwd	AGGTTCCGTCTTACTGGGTG	Vanheule et al. (2016)	Insertion 2
INS2-FLANK-rev	GCAGTACAAGCTACGATGGC	Vanheule et al. (2016)	Insertion 2
1285	GCGTCTCAGCTTCATCAAGGCAKCKAMTGAWTCG	Proctor et al. (2009)	*Tri1*
1292	CTTGACTTSMTTGGCKGCAAAGAARCGACCA	Proctor et al. (2009)	*Tri1*
pogo.fwd	AGACTCCGTACTGCCTTCAC	Vanheule et al. (2016)	*pogo* insertion
pogoabsent.rev	CTCCCCTGCAAAACATAGCC	Vanheule et al. (2016)	*pogo* insertion
pogopresent.rev	TATAGGGCTCTTTCAGGGGC	Vanheule et al. (2016)	*pogo* insertion

References:

Kerenyi, Z.; Moretti, A.; Waalwijk, C.; Olah, B.; Hornok, L. Mating type sequences in asexually reproducing fusarium species. *Appl. Environ. Microbiol.* **2004**, *70*, 4419-4423.
Parry, D.W.; Nicholson, P. Development of a pcr assay to detect Fusarium poae in wheat. *Plant Pathol.* **1996**, *45*, 383-391.
Vanheule, A.; Audenaert, K.; Warris, S.; van de Geest, H.; Schijlen, E.; Hofte, M.; De Saeger, S.; Haesaert, G.; Waalwijk, C.; van der Lee, T. Living apart together: Crosstalk between the core and supernumerary genomes in a fungal plant pathogen. *Bmc Genomics* **2016**, *17*, 670.
Proctor, R.H.; McCormick, S.P.; Alexander, N.J.; Desjardins, A.E. Evidence that a secondary metabolic biosynthetic gene cluster has grown by gene relocation during evolution of the filamentous fungus fusarium. *Molecular Microbiology* **2009**, *74*, 1128-1142.

Table S3. Isolates from additional Fusarium species collected for this study. "-" indicates that the year of isolation is unknown.

ID	Species	Location	Host	Year	Reference
1070	*F. culmorum*	Verrebroek, Belgium	soil	2011	this study
2702	*F. culmorum*	Zwalm, Belgium	wheat	2011	this study
2799	*F. culmorum*	Scy, Belgium	wheat	2011	this study
861	*F. culmorum*	Koksijde, Belgium	soil	2011	this study
888	*F. culmorum*	Poperinge, Belgium	soil	2011	this study
2321	*F. graminearum*	Ciney, Belgium	wheat	2010	this study
2322	*F. graminearum*	Ciney, Belgium	wheat	2010	this study
2415	*F. graminearum*	Bottelare, Belgium	wheat	2011	this study
2471	*F. graminearum*	Poperinge, Belgium	wheat	2011	this study
2472	*F. graminearum*	Poperinge, Belgium	wheat	2011	this study
2475	*F. graminearum*	Poperinge, Belgium	wheat	2011	this study
2598	*F. graminearum*	Zuienkerke, Belgium	wheat	2011	this study
2715	*F. graminearum*	Verrebroek, Belgium	wheat	2011	this study
8/1	*F. graminearum*	Germany	unknown	-	Jansen et al. (2005)

6133	*F. sporotrichioides*	Heverlee, Belgium	tobacco	1964	MUCL
113234	*F. langsethiae*	Norway	oats	-	CBS
2004/170	*F. langsethiae*	United Kingdom	wheat	2004	Imathiu et al. (2009)
2004/171	*F. langsethiae*	United Kingdom	wheat	2004	Imathiu et al. (2009)
041/11	*F. langsethiae*	United Kingdom	oats	2004	Imathiu et al. (2009)
2004/59	*F. langsethiae*	United Kingdom	oats	2004	Imathiu et al. (2009)
201086	*F. langsethiae*	Roverud, Norway	oats	2011	dr. Ingerd Hofgaard
201087	*F. langsethiae*	Roverud, Norway	oats	2011	dr. Ingerd Hofgaard
NRRL54940	*F. langsethiae*	Norway	oats	2011	dr. Ingerd Hofgaard
34988	*F. langsethiae*	Unknown	wheat	1992	MUCL

References:

Imathiu, S.M., Ray, R.V., Back, M., Hare, M.C., and Edwards, S.G. Fusarium langsethiae pathogenicity and aggressiveness towards oats and wheat in wounded and unwounded in vitro detached leaf assays. *European Journal of Plant Pathology* **2009**, *124*, 117-126.

Jansen, C., von Wettstein, D., Schafer, W., Kogel, K.H., Felk, A., and Maier, F.J. (2005). Infection patterns in barley and wheat spikes inoculated with wild-type and trichodiene synthase gene disrupted Fusarium graminearum. *Proceedings of the National Academy of Sciences of the United States of America* **2005**, *102*, 16892-16897.
